# Solving the two-decades-old murder case through joint application of ZooMS and ancient DNA approaches

**DOI:** 10.1007/s00414-022-02944-5

**Published:** 2023-01-10

**Authors:** Yang Xu, Naihui Wang, Shizhu Gao, Chunxiang Li, Pengcheng Ma, Shasha Yang, Hai Jiang, Shoujin Shi, Yanhua Wu, Quanchao Zhang, Yinqiu Cui

**Affiliations:** 1 School of Life Sciences, Jilin University, Changchun, 130012 China; 2 Max Planck Institute for the Science of Human History, 07745 Jena, Germany; 3 School of Pharmaceutical Sciences, Jilin University, Changchun, 130021 China; 4 Criminal Police Detachment, Qingdao Municipal Public Security Bureau, Qingdao, 266034 China; 5 Criminal Investigation Team, Jimo Branch, Qingdao Municipal Public Security Bureau, Qingdao, 266205 China; 6 Division of Clinical Research, First Hospital of Jilin University, Changchun, 130021 China; 7 Bioarchaeology Laboratory, Jilin University, Changchun, 130012 China; 8 School of Archaeology, Jilin University, Changchun, 130012 China

**Keywords:** Kinship identification, Cold-case investigation, Zooarchaeology by mass spectrometry, Ancient DNA, Whole-genome sequencing

## Abstract

**Abstract:**

Bones are one of the most common biological types of evidence in forensic cases. Discriminating human bones from irrelevant species is important for the identification of victims; however, the highly degraded bones could be undiagnostic morphologically and difficult to analyze with standard DNA profiling approaches. The same challenge also exists in archaeological studies. Here, we present an initial study of an analytical strategy that involves zooarchaeology by mass spectrometry (ZooMS) and ancient DNA methods. Through the combined strategy, we managed to identify the only biological evidence of a two-decades-old murder case — a small piece of human bone out of 19 bone fragments — and confirmed the kinship between the victim and the putative parents through joint application of next-generation sequencing (NGS) and Sanger sequencing methods. ZooMS effectively screened out the target human bone while ancient DNA methods improve the DNA yields. The combined strategy in this case outperforms the standard DNA profiling approach with shorter time, less cost, as well as higher reliability for the genetic identification results.

**Highlights:**

• The first application of zooarchaeology by mass spectrometry technique in the forensic case for screening out human bones from bone fragment mixtures.

• Application of ancient DNA technique to recover the highly degraded DNA sequence from the challenging sample that failed standard DNA profiling approaches.

• A fast, sensitive, and low-cost strategy that combines the strengths of protein analysis and DNA analysis for kinship identification in forensic research.

**Supplementary Information:**

The online version contains supplementary material available at 10.1007/s00414-022-02944-5.

## Introduction

Bioanalytical chemistry has played a more important role in the field of forensic research since the first introduction of DNA profiling in the 1980s [1]. The development of strategies which are rapid, low-cost, and sensitive for challenging samples will undoubtedly be the trend of future molecular forensic research [2, 3]. And various techniques have been continuously upgraded for identifying different biological materials in forensic investigations. In recent years, proteomic evidence (mainly from bodily fluids and skin remains) has also been used in the criminal justice community [4–6]. The rapid development of mass spectrometry technology allows trace amount protein/peptide determination, showing great potential in forensic practice.

Obtaining robust evidence from severely degraded skeleton remains is still a major challenge in forensic practices, especially when the sample is a mixture of undiagnostic bone fragments. With DNA analysis only, more effort is needed on taxonomical identification than on biometrics recognition of individual. However, this kind of samples has commonality with archaeological bone materials, in terms of the specimen’s preservation and mixed property. It is worth referring to archaeological approaches for a high-efficient forensic solution. Recent advances in ancient DNA technology, particularly the application of next-generation sequencing (NGS), enable us to recover severely damaged DNA sequence from even ancient samples; thus, it could become an applicable tool for genetic analysis in forensic cases [7, 8].

Zooarchaeology by mass spectrometry (ZooMS) is a proteomic approach based on the collagen peptide mass fingerprinting (PMF) technique, providing taxonomic information through the detection of tryptic peptides of two type I collagens via matrix-assisted laser desorption ionization time-of-flight mass spectrometry (MALDI-TOF-MS). Collagen is phylogenetically informative and it can persist for longer periods than DNA [9–11]. Since invented [12], ZooMS has been used in diverse research fields including archaeology and paleontology, ecology and conservation, as well as cultural heritages [13], applied on a wide range of collagenous materials such as leather, ivory, and parchment [14–17], but mostly on bones. And the technique lends itself particularly well to being utilized for the large-scale taxonomic investigations of faunal assemblages as well as identification of animal remains or products lacking diagnostic features for traditional zooarchaeological determinations. Compared to the DNA taxonomic approaches, ZooMS has advantages including simple procedures, high-throughput, and low detection cost. Previous studies have shown that this technique could provide a quite high identification success rate (>95%) for the analysis of archaeological samples from Late Pleistocene [18–21]. The ZooMS method could no doubt provide reliable taxonomic information for mixed forensic samples before performing the DNA profiling analysis.

In this case, we creatively combined two mature techniques from different fields to solve a cold case that seems to have reached a dead end. Firstly, the ZooMS method is performed to screen out human bones from a mixture of bone fragments, with low cost and short time; then ancient DNA technology is used to recover the highly degraded DNA sequence. Following the analysis of the ancient genome to assess the sex and probable geographic origins, together with the kinship possibility calculation between the individual and the putative parents, we finally managed to provide vital biological evidence for the case.

## Case history

In 2002, a 9-year-old boy in Qingdao, Shandong Province, went missing after school. The parents had searched across China for years in vain. After 20 years, the police finally locked the suspect, who had confessed guilty of murdering the boy, burying the body in his own yard filled with domestic wastes (including animal bones), and moved the body to the cropland which is unable to be located several years after the murder. Therefore, efforts to identify the victim’s remains ran into difficulties; only a few undiagnostic bone fragments were found in the yard deposit and most of them are less than 2 cm in size (too small to be identified morphologically) and extremely porous and fragile. Routine forensic identification approaches including STR testing was conducted on some of the bones but failed to retrieve any valuable genetic information, probably due to the high degradation level of the DNA molecules in the samples. Since it is of great urgency to identify whether there was any bone(s) that belongs to the victim, a mixture of 19 bone fragments was finally transferred to the ancient DNA laboratory at Jilin University.

## Materials and methods

### Samples

**Fig. 1:**
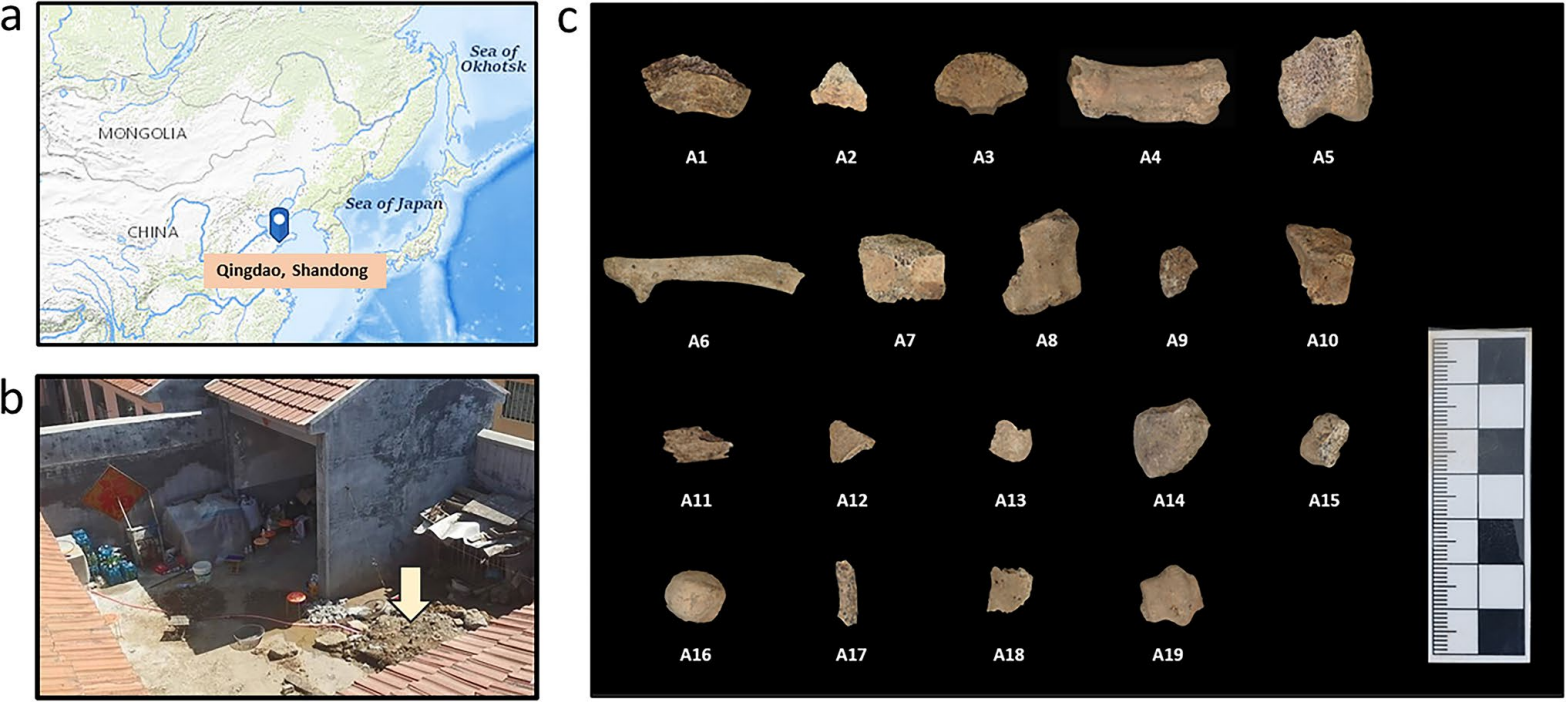
Location of the burial site and the bone fragments analyzed in this study. **a** Location of Qingdao City, where the murder case took place. The base map was obtained from the USGS National Map Viewer, public domain (http://viewer.nationalmap.gov/viewer/). **b** The burial site in the suspect’s yard. **c** Nineteen bone fragment samples excavated at the burial site, and the test numbers are marked at the bottom of each sample

The responsible justice department provides all the samples and authorizes all protein and DNA testing. Nineteen bone samples were collected and photographed, as shown in Fig. 1. The bones were small, from 0.9 to 4.4 cm in length, bearing no morphological feature. The surface of some bones was damaged by postmortem erosion in the humic soil. To remove any contaminants attached to the surface of the bone, a small area was sandblasted. Around 50 mg of bone chip was sub-sampled from each sample for ZooMS. The amount for the following DNA analysis is also about 50 mg, in powder. In our study, the blood samples from the putative mother and father (pM and pF) were also collected on the FTA cards as references.

### Taxonomic identification by ZooMS

The collagen in the bone samples was first extracted following the established acid-insoluble protocols [22]: samples were demineralized in 500 μL 0.5 M HCl for 6 h at 4 °C until the bone chips became spongy. The supernatant was then removed and the chips were rinsed 3 times using 0.5 M NH_4_HCO_3_ until a neutral pH was reached. The chips were incubated at 65 °C for 1 h, in 100 μL of 50 mM NH_4_HCO_3_. Following incubation, 50 μL supernatant was collected and was digested with 0.5 μg trypsin at 37 °C for 18 h. One microliter of 5% trifluoroacetic acid (TFA) was added to end the digestion. The digested samples were concentrated and desalted using C18 ZipTips, then washed with 200 μL 0.1% TFA and eluted with 50 μL 50% ACN/0.1% TFA (v/v). One blank was analyzed alongside samples as a negative control. Then MALDI-TOF-MS analysis was carried out to obtain the collagen peptide mass spectra for all samples (Supplementary Text), and species were identified using previously published type I collagen peptide markers from reference spectra [23].

### DNA analyzing

The only bone fragment identified as human bone by ZooMS (A14, see below) was then conducted by DNA analysis. The genetic relationship identification by routine STR test failed, which might be due to the high degradation level of the DNA molecules in the bone fragment. Therefore, strict ancient DNA protocols were applied during DNA exaction, pre-PCR, and DNA library construction of the bone sample [24], while DNA extraction and library preparation for blood samples from pM and pF were undertaken in the modern genetic laboratory. And both shotgun sequencing and Sanger sequencing were performed on all three samples.

### DNA extraction, library preparation, and shotgun sequencing

DNA extraction of A14 was performed in a laboratory for ancient DNA located in the College of Archaeology, Jilin University, and treated as described by Li et al. [25] (Supplementary Text). DNA extraction and library preparation for samples from pM and pF were undertaken by TruePrep® Flexible DNA Library Prep Kit for Illumina (Vazyme, China) in the modern genetic laboratory in the School of Life Sciences, Jilin University, and all the experiments were performed only after genome profile was obtained for bone fragment to avoid the possibility of contaminating the “cold case” remains with the modern reference DNA sample. All the libraries were sent to sequence on Illumina HiSeq X Ten platform.

### Authenticity control

In order to evaluate possible contamination of A14 and verify the authenticity of the results, we computed the proportion of C-to-T deamination errors at both the 5′ and 3′ ends of the sequencing reads to evaluate the postmortem damage patterns and then examined mtDNA contamination using Schmutzi [26], an approach that calls endogenous DNA based on the deamination patterns and computes the contamination rate by comparison to a set of known contaminants.

### Sanger sequencing

With over 99% accuracy, the Sanger sequencing method remains the “gold standard” for individual identification or kinship testing. To validate mtDNA variants that firstly identified through NGS, we further perform Sanger sequencing targeting the mtDNA hypervariable region I (HVR-I). Two sets of overlapping primers were used to amplify the mtDNA HVR-I between positions 16035 and 16409, and PCR amplifications were done for A14 as described by Li et al. [25], but increasing the number of PCR cycles to 40. Amplification products were sequenced directly using the Sanger sequencing method (ABI PRISM 3130). The amplification and sequencing from bone extract were repeated twice. Extraction blanks and PCR negative controls were carried out for each PCR experiment.

### Genomic data processing

The Sanger sequencing result was converted to mtDNA sequence information by Chromas (http://technelysium.com.au/wp/chromas/); the consensus mtDNA sequence obtained from multiple overlapping PCR amplifications was compared to those obtained from pM and pF. For the processing of the shotgun results, the raw fastq files from Illumina platform were processed in EAGER v1.92.50 program [27], an automated computational pipeline specially designed for ancient DNA data processing, which is described in detail in Supplementary Text. The biological sex of A14 was assessed by computing the ratio of X chromosome derived shotgun sequencing data to the autosomal coverage. We measured the rate of damage using mapDamage v2.0.6 [28].

### Genetic structure analysis

The uniparental haplogroups of A14, pM, and pF were assigned, and the procedure is described in Supplementary Text. Briefly, the mtDNA consensus sequences were generated using the Geneious software [29], and then determined their mtDNA haplogroups using HaploGrep2 [30]. The male Y chromosome haplogroup was determined by examining a set of positions on the 25,660 diagnostic positions of the ISOGG database (https://isogg.org/), and assigned the final haplogroups by the most downstream derived SNPs. The whole-genome data of three samples in this case was compared to modern populations in the Affymetrix Human Origins (HO) public dataset [31, 32] or the high-coverage Simons Genome Diversity Project [33, 34] and the final dataset consists of 593,124 autosomal SNPs. The genetic affinities of our samples with present-day Asian populations were assessed by principal component analysis (PCA) and outgroup f3 statistics (see Supplementary Text for more details). The PCA was carried out using the “lsqproject” options in the smartpca program [35]. We also implemented outgroup f3 statistics using qp3Pop (v435) program in the ADMIXTOOLS package [36] with Mbuti population from Central Africa as an outgroup, and the f3-statistics were performed on the 1240k dataset.

### Genetic relatedness estimation between A14 and the putative parents

Because of the relative low coverage of DNA data from the bone fragment, we applied pairwise mismatch rate (PMR) methods to determine the genetic kinship between A14 and his putative relatives [37]. The PMR approach was designed specially to estimate kinship of ancient samples, by calculating the pairwise mismatch rate of haploid genotypes across autosomal SNPs. The PMR value for each pair of individuals was defined by dividing the number of SNP sites for which two individuals have different alleles sampled by the total number of sites covered in both individuals. In general, the PMR of the identical individuals (*r* = 1) should be half of that between the unrelated individuals (*r* = 0, identified as the population baseline, no inbreeding). Likewise, the PMR for first- (*r* = 0.5) and second-degree relatives (*r* = 0.25) should be 3/4 and 7/8 of the baseline, and more details were described in Supplementary Text.

## Results

### ZooMS results

**Fig. 2:**
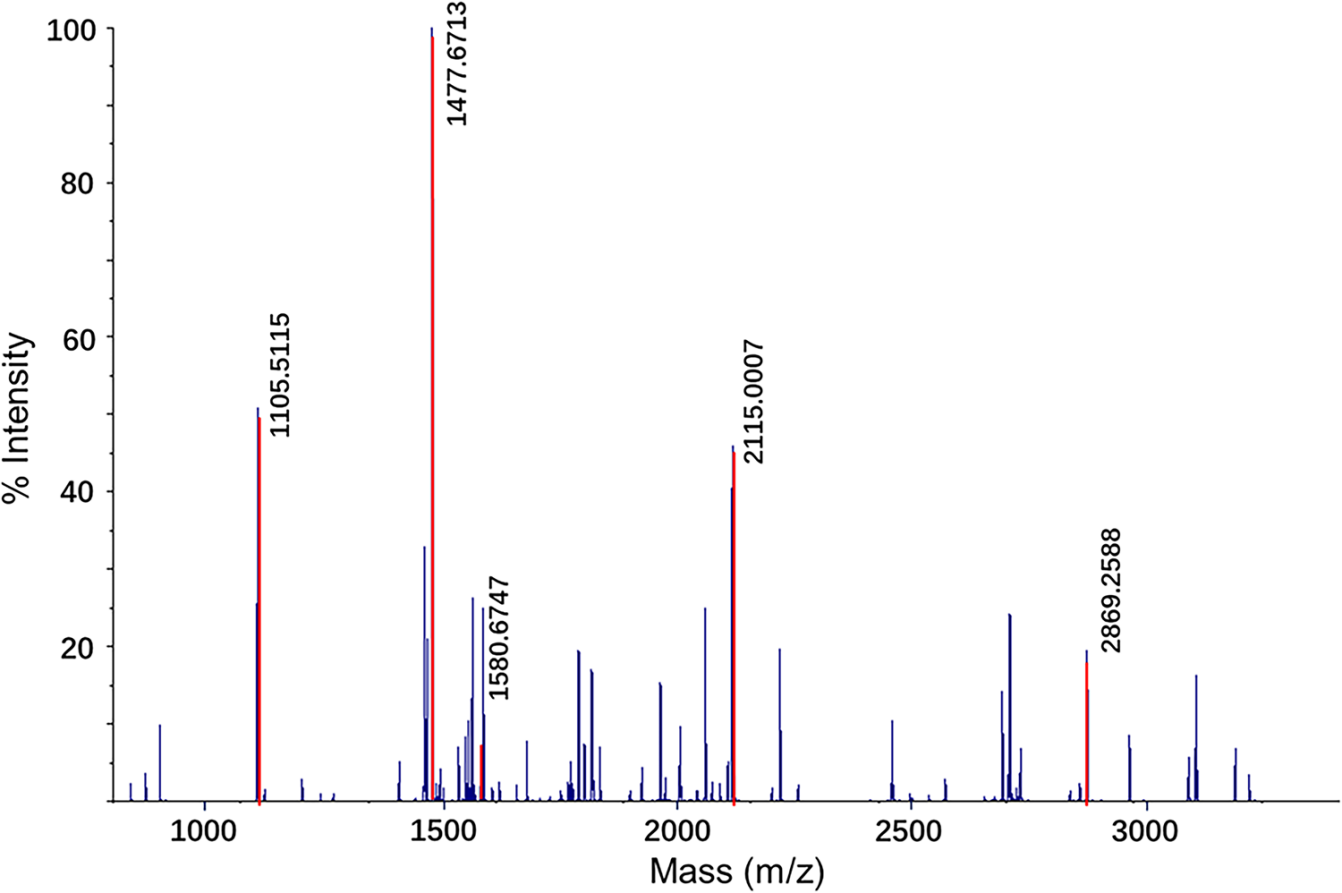
Spectrum of A14 obtained by the MALDI-TOF mass spectrometer. Five peptide markers assigned A14 to *Homo sapiens* are colored in red

After comparing the spectra generated from MALDI-TOF-MS against the published reference [18, 23], taxonomic information of the samples was obtained. Eighteen out of the 19 samples were identified and fortunately, one of the samples, A14, was identified as human (*Homo sapiens*) (Fig. 2). Among the rest 17 samples, one was an avian sample (A6) which could be a chicken or a duck, and the other 16 samples were all identified as pig (*Sus scrofa*). The results are consistent with the fact that they were from a domestic waste deposit. For sample A11, only 3 markers (*m*/*z* 1105, *m/z* 1453, and *m*/*z* 2820) were detected, which is not enough for more accurate taxonomic identification, indicating a higher level of degradation. The blank control returned negative result, and no cross-contamination was observed. Details of the ZooMS results are shown in Supplementary Fig. S1 and Table S1. The ZooMS taxonomic identification was completed in 3 days, with the cost less than 10 dollars per sample.

### DNA analysis results

#### The authenticity of the genome data

We applied strict procedures to minimize exogenous DNA contamination following the ancient DNA standard. During the experiment process, the negative extraction and amplification controls were free of contamination, and the multiple sequencing results were consistent, including twice Sanger sequencing and shotgun sequencing. Through analyzing the characteristics of genomic library reads, we also observed that the A14 exhibited postmortem chemical damage signatures of DNA molecular, such as small average sequence size with 75 bp, and the nucleotide misincorporation patterns at the 3′- and 5′- ends of the DNA sequences (Table 1, Supplementary Fig. S2). Meanwhile, the sequence reads from A14 showed a low level of contamination for mtDNA (0.5%). The results proved our previous degradation assessment of the sample, and verified the authenticity of A14 data as well. Our sex determination results also show the A14 was from a male individual.

**Table 1:** High-throughput sequencing result of DNA library of A14, pM, and pF

Sample name	Endogenous DNA (%)	Mean coverage	SNP	Average fragment length	mt-hg	Y-hg	mt-contamination
A14	19.776	0.0548	50021	75.08	D4j3	O2a2b1a1a5	0.005
pM	94.763	1.0067	597100	163.11	D4j3	-	-
pF	94.272	0.8785	546202	170.4	D4g2a1	O2a2b1a1a5	-

#### Genetic analyses of A14

**Fig. 3:**
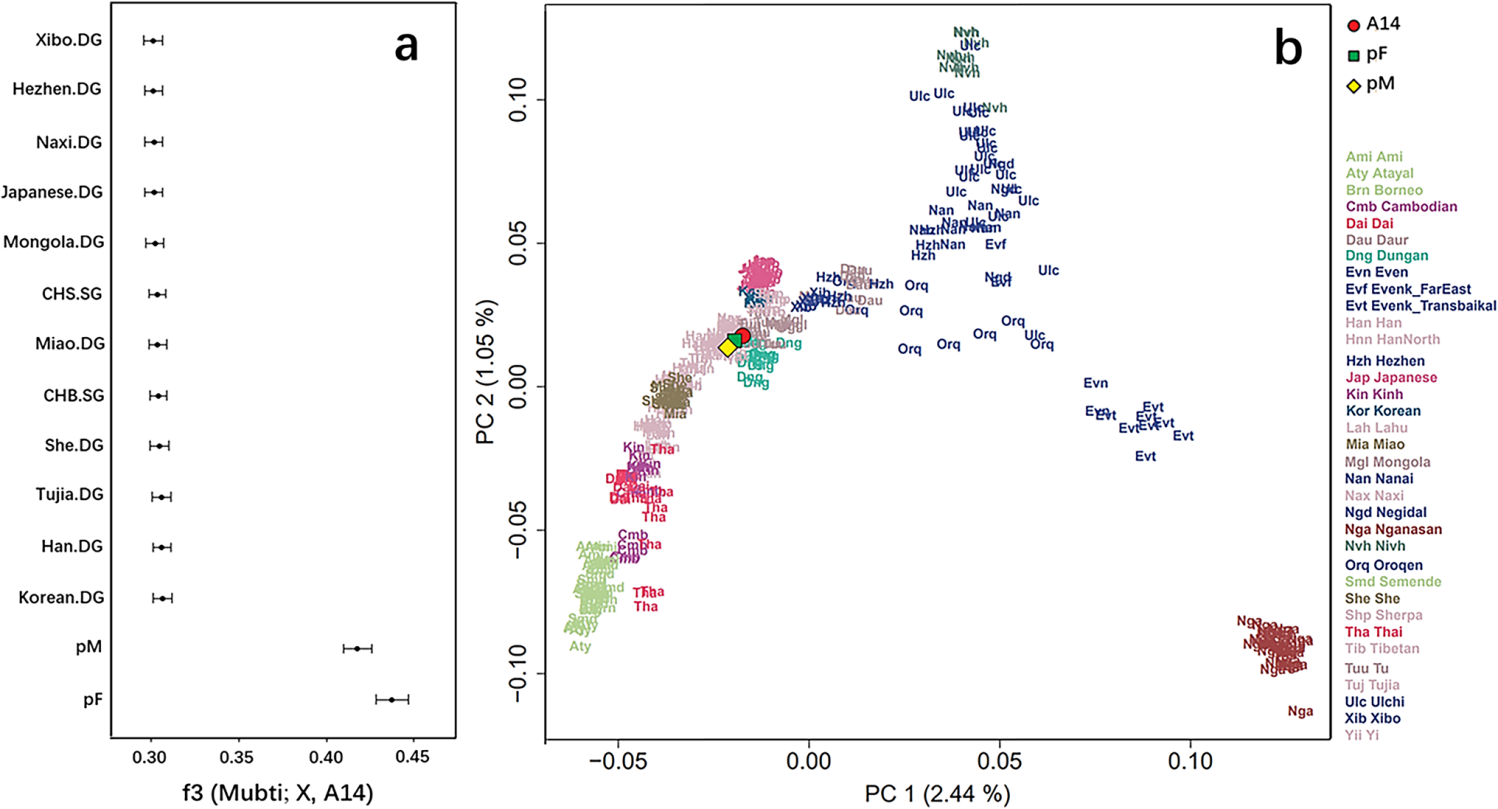
The genetic profile of A14 by outgroup f3 and PCA. **a** The top 14 populations (including the putative parents) sharing the highest amount of genetic drift with A14 measured by f3 (Mbuti; X, A14). **b** Principal component analysis of A14 and the putative parents projected onto present-day Asian populations

We successfully extract endogenous DNA from A14 and the DNA library was sequenced to a low coverage with 0.044×. To characterize the genetic profile of the A14, we implemented principal component analysis (PCA) of present-day Asian people and A14 genome. The results show that the genetic distributions of modern people are consistent with the geographic locations in the PCA plot [38]. We found that the A14 is falling in the group of modern Han and clustered with the putative parents (Fig. 3b). The observation from the PCA plot was further confirmed by the outgroup f3 statistics in the form of (Mbuti; X, A14), where X was represented by worldwide populations; the result showed significant allele sharing between A14 and the putative parents, followed by Eastern Asia populations, such as Han, Korean, and Tujia (Fig. 3a), indicating a much closer genetic affinity of A14 with the putative parents.

#### Uniparental and autosomal genetic kinship analysis with putative parents

We retrieved almost complete mtDNA sequence (99.5%) for A14, with an average coverage of 10.16-fold, which were further assigned to an explicit haplogroup of D4j3 which is the most prevalent haplogroup in the modern East Asian populations [39]. To confirm the results of NGS, we used two sets of overlapping primers to amplify the HVR of the mtDNA control region, and used the Sanger sequencing method to obtain the HVR-I sequence, which contains the mutant motif 16184-16223-16311-16362. Sanger sequencing results were consistent with the mitochondrial genome obtained by NGS. The mtDNA genome reconstructed from the shotgun genome data of pM produced the same mtDNA profile as A14. But a different profile was obtained from pF, which belonged to haplogroup D4g2a1 (Table 1, Supplementary Table S2). In contrast, the A14 was assigned to Y chromosome haplogroup O2a2b1a1a5 the same as pF (Table 1, Supplementary Table S3), which is a widespread lineage in modern Northeastern Asians such as Sino-Tibetan-speaking populations [40]. Uniparental results showed probable maternal and paternal kinship between A14 and pM, as well as A14 and pF, independently.

**Fig. 4:**
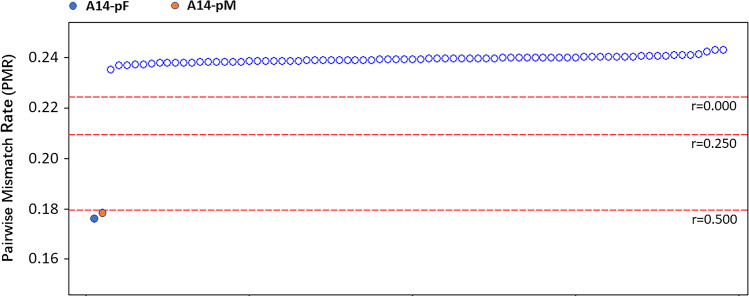
Pairwise mismatch rate (PMR) between A14 and the putative parents. Baseline was generated by PMR calculated within northern Han individuals (*n* = 10)

To estimate the genetic relatedness between A14 and the two putative parental samples at a finer scale, the degree of genetic relatedness between individuals from autosomes was determined. We calculated PMR from haplotype genotyping of the “1240k” panel using a special method designed for ancient DNA [41]. The overlapping SNPs pairs between A14 and the test samples reached over 20,000, which is sufficient to avoid the artificial bias caused by the high deletion rate of A14. The PMR value between pM and pF was 0.238, which is similar to the value from unrelated pairs of modern northern Han. This suggested they had no close relatedness with each other, which is consistent with their self-reported genetic background. We therefore treated it as the baseline value, together with those obtained between pairs of unrelated modern Han. The PMR values for A14-pM and A14-pF were 0.178 and 0.176, respectively, roughly 1/2 of the baseline value (Fig. 4), suggesting that A14 shares first-degree relatedness with them.

## Discussion

### The retrieved genetic profile of A14

Through the joint application of ZooMS and ancient DNA approaches, we efficiently screen out the only human bone, A14, among 19 bone fragments, and retrieve the genetic profile of the target sample, as follows: the sample is from a human male skeleton, both the uniparental haplogroup distribution and the genome composition of A14 indicated a geographic origin in East Asia together with pM and pF. From uniparental genetic analyses, we found that A14 was potentially maternally related to pM, and paternally related to pF. Autosomal analysis revealed first-degree relatedness of A14 with these two individuals separately, and the genetic data of the two putative parents proved their unrelatedness, from maternal and genomic perspectives, a result consistent with the de facto relationship between the two individuals. Altogether, our combined test results confirmed that the remains A14 most likely belong to the missing 9-year-old boy.

### Combined ZooMS and ancient DNA as a promising approach in the forensic practice

Bones are one of the most common biological types of evidence in forensic cases. Due to the preservation conditions or special underground environment, sometimes only a mixture of poorly preserved bones is recovered at the crime scene. The specimens could be fragmentary or lacking diagnostic morphological features. In practice, doing standard DNA profiling on every sample in the mixture is possible but not the most efficient solution. ZooMS, as a taxonomic method, is able to identify the crime-relevant species (like *Homo sapiens*), from lots of irrelevant remains (like bones belonging to *Sus scrofa* and Aves in this case). Another difficulty is that the DNA molecules maybe severely degraded because of the humic environment, resulting in an average length of DNA fragments less than 80 bp. The length is similar to the archaeological samples, making it difficult to perform routine STR tests and other forensic methods commonly used for genetic identification.

In this case, our aim was to obtain the biological evidence of a victim. The bones (*n* = 19) in mixture were highly fragmented and degraded. We designed a two-step analytical strategy in our study. ZooMS is used in the first step to screen the human bone fragments from the mixture, and the whole process only took three workdays. With a relatively low cost, one human bone (A14) was successfully identified. The second step involved profiling methods from the ancient DNA field, and combining the reliable accuracy of Sanger sequencing with the high-throughput nature of NGS, we obtained authentic genomic sequences of the victim, which passed the uniparental genetic marker analysis and the relativeness calculation, matched to the putative parents.

To sum up, through introducing the state-of-the-art technologies in archaeology into the forensic practice, we identified and confirmed the bone fragment of the victim boy with high efficiency. This study provides new evidence for solving cold cases and highlights the enormous potential of multidisciplinary techniques applied in forensic study for crime solving and justice.

## Supplementary Information

ESM 1: (DOCX 3366 kb)
