# Supplementary material for: Solving the two-decades-old murder case through joint application of ZooMS and ancient DNA approaches
Source: Int J Legal Med. 2023 Jan 10;137(2):319–27. doi: 10.1007/s00414-022-02944-5 (PMC9902420; doi:10.1007/s00414-022-02944-5)
Supplement: Supplementary file 1 — (DOCX 3366 kb) [file 414_2022_2944_MOESM1_ESM.docx]

**International Journal of Legal Medicine**

**Supplementary Information for**

Solving the two-decades-old murder case through joint application of ZooMS and ancient DNA approaches

Yang Xu^a^, Naihui Wang^b^, Shizhu Gao^c^, Chunxiang Li^a^, Pengcheng Ma^a^, Shasha Yang^a^, Hai Jiang^d^, Shoujin Shi^e^, Yanhua Wu^f^, Quanchao Zhang^g,h^, Yinqiu Cui^a,^^g^*

^a^ School of Life Sciences, Jilin University, 130012, Changchun, China;

^b^ Max Planck Institute for the Science of Human History, 07745, Jena, Germany;

^c^ School of Pharmaceutical Sciences, Jilin University, 130021, Changchun, China;

^d^ Criminal Police Detachment, Qingdao Municipal Public Security Bureau, 266034, Qingdao, China;

^e^ Criminal Investigation Team, Jimo Branch, Qingdao Municipal Public Security Bureau, 266205, Qingdao, China;

^f^ Division of Clinical Research, First Hospital of Jilin University, 130021, Changchun, China;

^g^ Bioarchaeology Laboratory, Jilin University, 130012, Changchun, China;

^h^ School of Archaeology, Jilin University, 130012, Changchun, China.

**Corresponding author**

* Yinqiu Cui, School of Life Sciences, Jilin University, 130012, Changchun, China, +86 13604337044, cuiyq@jlu.edu.cn

**This file includes:**

**Supplementary Text**

MALDI-TOF-MS analysis.

DNA extraction, library preparation and shotgun sequencing for A14.

Shotgun data processing.

Uniparental haplogroup assignment.

Population genetic structure analysis.

Genetic relatedness analysis.

**Supplementary Figures**

Fig. S1 MALDI-TOF spectra of the bone fragments.

Fig. S2 DNA damage level of A14 measured by the rate of cytosine deamination-based misincorporation of bases as a function of position on reads.

**Supplementary Tables**

Table S1 Taxonomic identification results of 19 samples by ZooMS.

Table S2 Results of mtDNA HVR fragments extracted from bone remain of A14 and blood samples of the putative parents.

Table S3 Results of Y chromosome haplogroup analysis of A14 and pF.

**Supplementary References**

**MALDI-TOF-MS analysis**

To prepare peptide samples for MS analysis, 0.5 µL of the resulting elution was spotted with an equal volume of α -cyano-4-hydroxycinnamic acid solution (10 mg/mL in 50 % ACN/0.1 % TFA (v/v)) on an Opti-TOF 384 MALDI plate insert (AB SCIEX, USA) prior to the analysis. Each sample or blank were spotted in triplicate. MALDI-TOF-MS analysis was carried out on a 5800 MALDI-TOF/TOF mass spectrometer (AB SCIEX, USA) coupled with a 355-nm Nd-YAG laser. The laser energy was adjusted to 4000, and all MS spectra were acquired in batch using the reflector detection in the positive ionization mode. Calibration was performed using a 6-peptide calibration mixture (Tube PN: 4368762, AB SCIEX, USA) to ensure mass accuracy within m/z 0.1. Mass spectra files were processed using the official data analysis software for MALDI-TOF/TOF mass spectrometer (AB SCIEX, USA) -Data Explorer version 4.3. Processing of raw spectra was conducted in Data Explorer with a peak picking algorithm that used a signal to noise ratio of 70.

**DNA extraction, library preparation and shotgun sequencing for A14**

DNA extraction of A14: Bone powder (50 mg) were incubated in a 3 mL solution containing 0.45 M ethylene diamine tetraacetic acid, 0.5 % SDS and 0.7 mg mL-1 proteinase K at 50 ℃ in a shaker (220 rpm/min) for 24 h, DNA was extracted using the QIAquick PCR Puriﬁcation Kit (Qiagen, Hilden, Germany) according to the manufacturer’s protocol. Two separate libraries were prepared from 30 μL bone DNA extract as described in our previously published paper [1], except that 1:10 diluted adapter was applied to the ends of DNA fragments during ligation. The quality and concentration of these two libraries and one library negative control were determined on an Agilent Bioanalyzer 2100 and multiplex shotgun sequencing was carried out using Illumina HiSeq X Ten platform.

**Shotgun data processing**

For the processing of the shotgun results, the raw fastq files from Illumina platform were processed in EAGER v1.92.50 program, an automated computational pipeline specially designed for ancient DNA data processing [2]. Specifically, in EAGER, Illumina Adapters were trimmed from sequencing data with AdapterRemoval v2.2.0 [3] and read length shorter than 30bp were discarded. The trimmed data was then mapped to the human reference genome (GRCh37) using BWA 0.7.12 with ‘-n 0.01’ and ‘-l 1024’ to allow for more mismatches and to disable the seeding. The duplicated reads were then removed with dedup v0.12.2 [2] and sequences with a mapping quality of ≥ 30 are retained using SAMtools [4]. Finally, we randomly called genotype for a SNP from trimmed reads with high-quality base (Q > 30) that implemented using pileupCaller (<https://github.com/stschiff/sequenceTools>).

**Uniparental haplogroup assignment**

To determine the mtDNA haplogroup, we first aligned the adapter trimmed reads to the revised Cambridge Reference Sequence (rCRS; NC_012920.1) and removed low-quality sequences (-q30). Next, we generated the mtDNA consensus sequences of our ancient individuals using the Geneious v11.1.3 (<https://www.geneious.com/>) and then assigned their mtDNA haplogroups using HaploGrep272.

We determined the male Y chromosome haplogroup by examining a set of positions on the 25,660 diagnostic positions on the ISOGG database (<https://isogg.org/>) and assigned the final haplogroups by the most downstream derived SNPs.

**Genetic structure analysis**

Principal components analysis (PCA), is a statistical method commonly used in population genetics to identify structure in the distribution of genetic variation across geographical location and ethnic background. When PCA was performed to low coverage data such as ancient genomic data, we usually construct the Eigenvectors firstly from the high-quality set of modern samples in the HO set, and then project the ancient or low coverage samples onto these Eigenvectors. This allows our A14 with as few as 50,000 SNPs to project into correct location of PCA plot (compare with ~600,000 SNPs for HO samples). In this case, we performed PCA for the genomic data of three samples as implemented in the smartpca v16000 in the Eigensoft v7.2.1 with default parameters, and Shrinkmode: YES and lsqproject: YES options to minimize bias due to high missing rate.

The outgroup f3-statistics measure allele frequency correlations between populations to understand population relationships. In this case, we calculated f3-statistics were using the qp3Pop (v435) programs in the ADMIXTOOLS v5.1 package using default parameters [5].

**Genetic relatedness analysis**

Genetic relatedness was estimated by calculating pairwise mismatch rate between each pair of individuals. The pairwise mismatch rate provides an indication of close [genetic relationships](https://www.sciencedirect.com/topics/biochemistry-genetics-and-molecular-biology/molecular-phylogeny), such as identical individuals/twins, first and second degree relatives. In this study, we tested relatedness among our three individuals by calculating PMR of haploid genotypes across autosomal SNPs in the 1240K data set, following the idea present by Kennett et al. [6]. PMR between unrelated individuals has a baseline value, which was obtained by estimating by the empirical distribution of PMR for multiple individuals, for example, ten unrelated modern northern Han individuals in this case. PMR values between A14 and pM, A14 and pF, pM and pF were calculated to finally estimate the genetic relatedness between A14 and the two putative parental samples. Notably, pairwise mismatch rate between two samples from the same individual (r=1) is expected to be a half of that between two unrelated individuals (r=0). Likewise, PMR for the first-degree relative pair (r=0.5) is expected to be three-quarters of the baseline. In general, pairwise mismatch rate is a linear function of the coefficient of relationship. More detailed description of the method can be found in the Supplemental Materials of Jeong et al.[7]


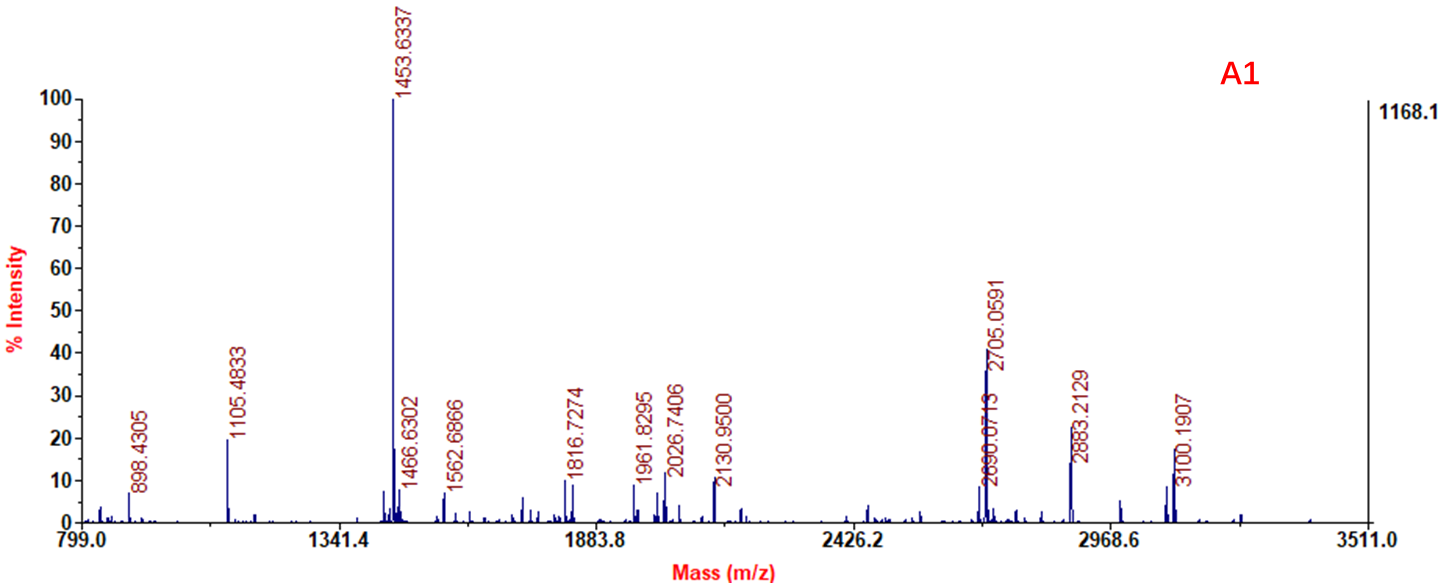

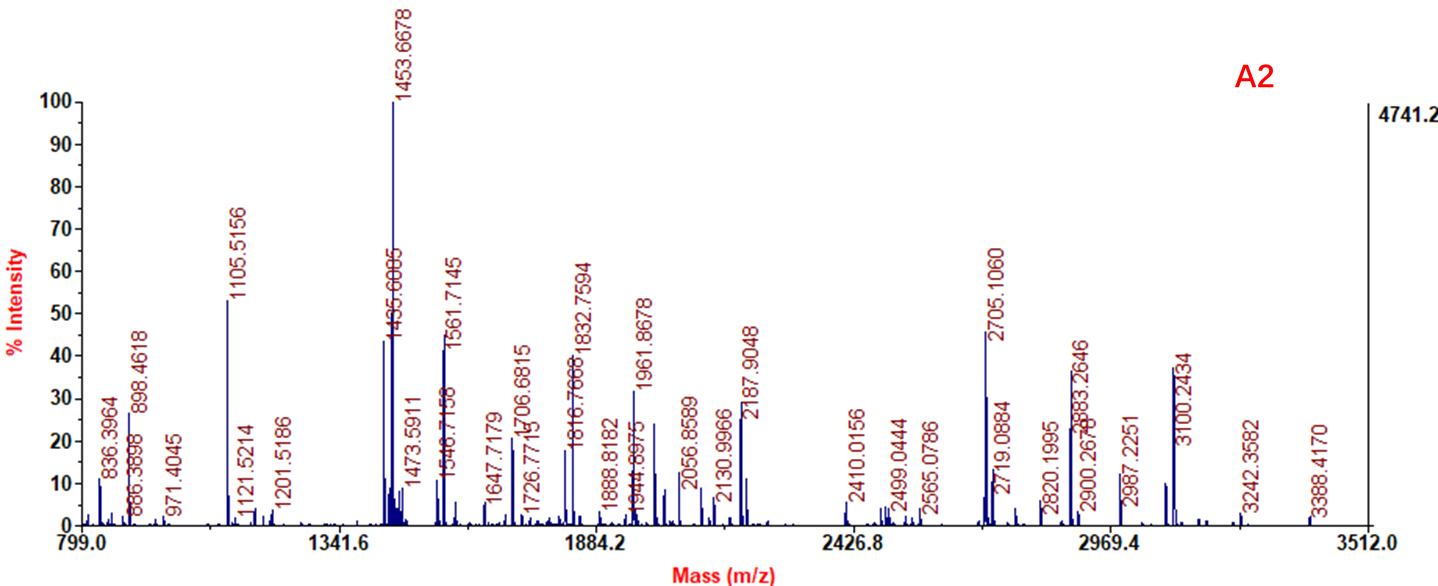

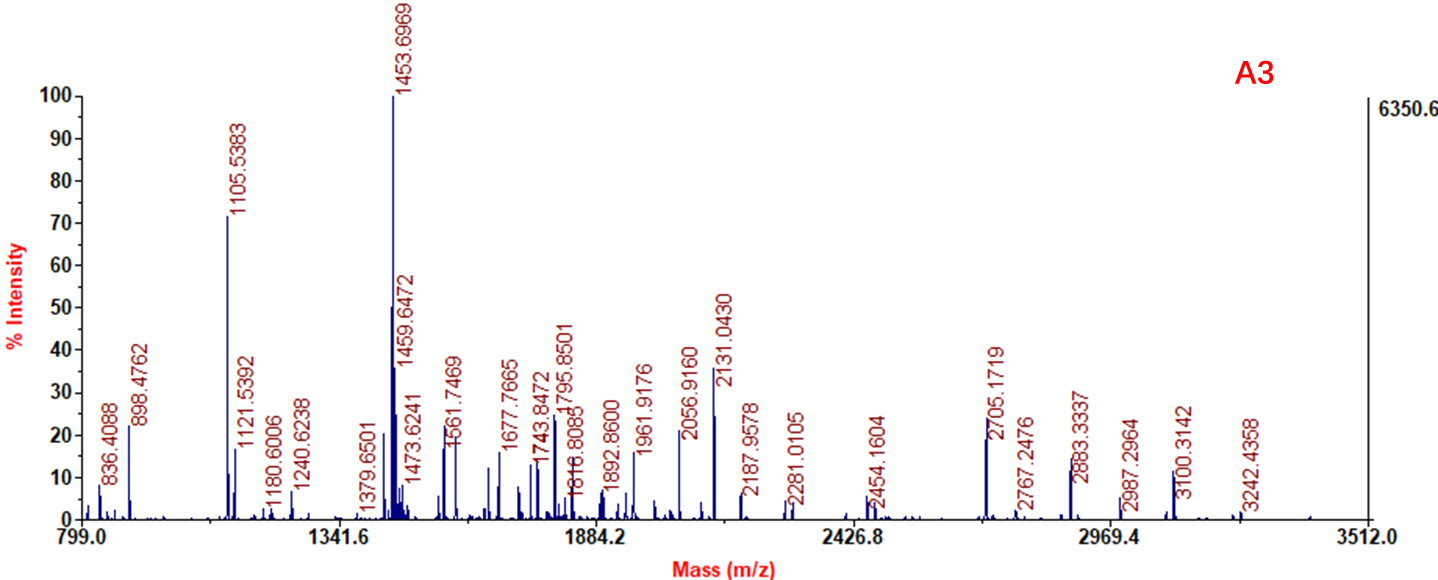

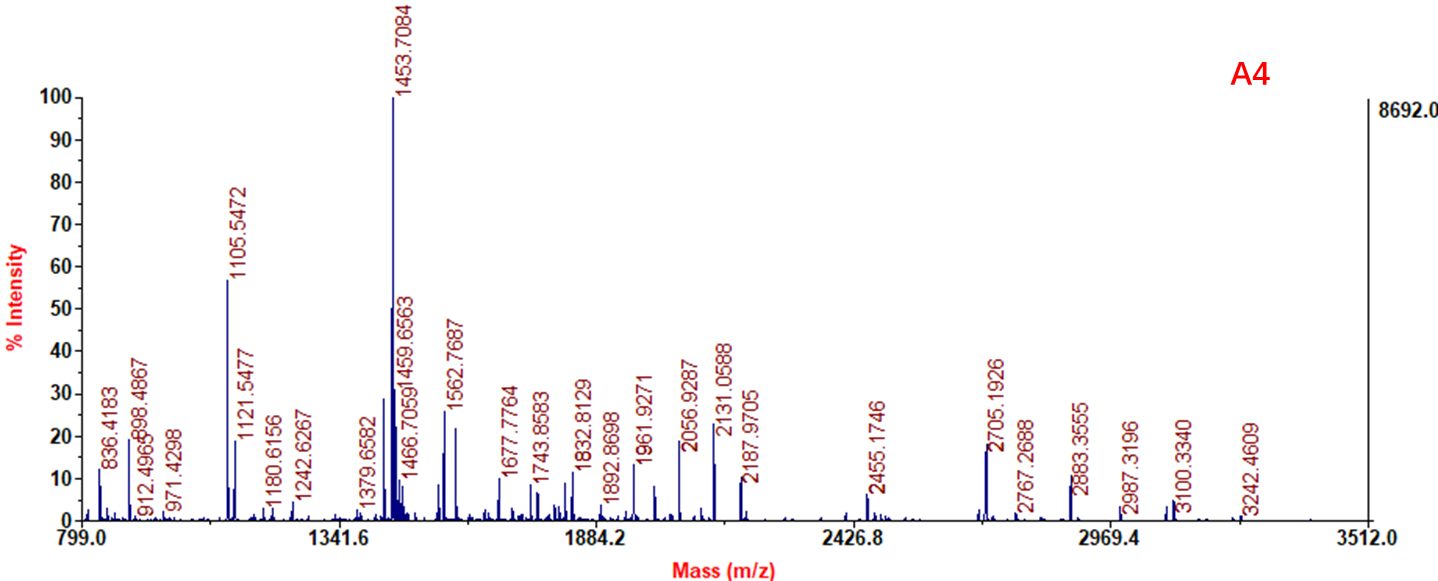

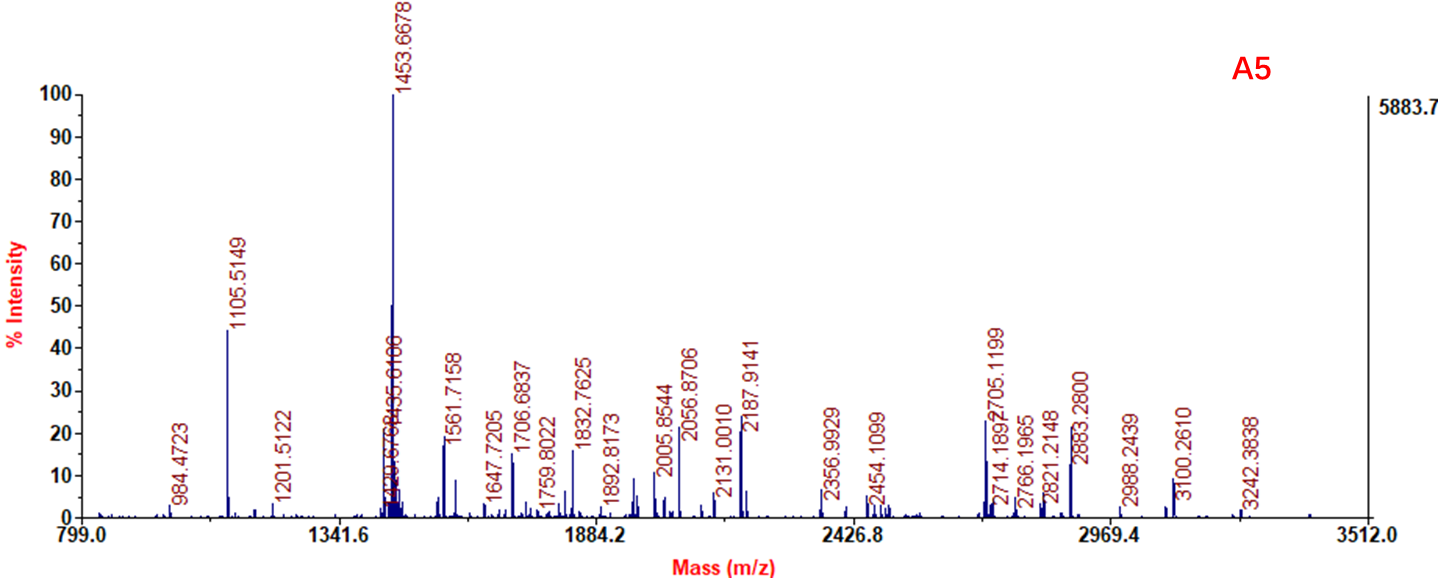

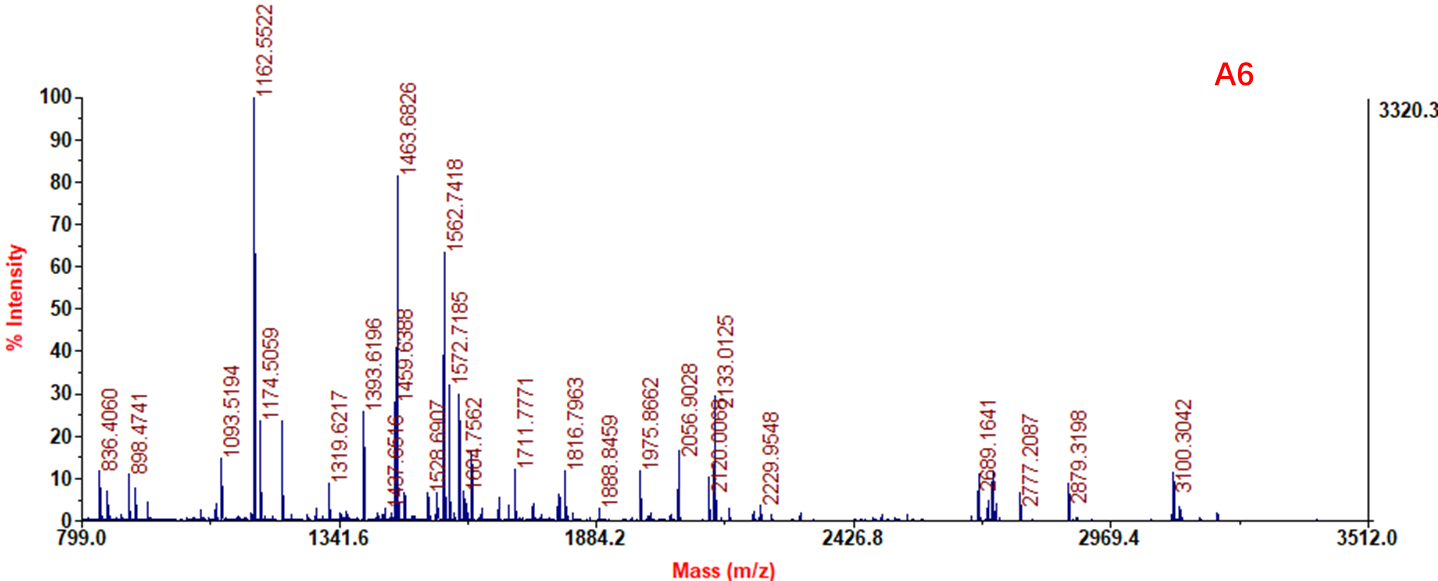

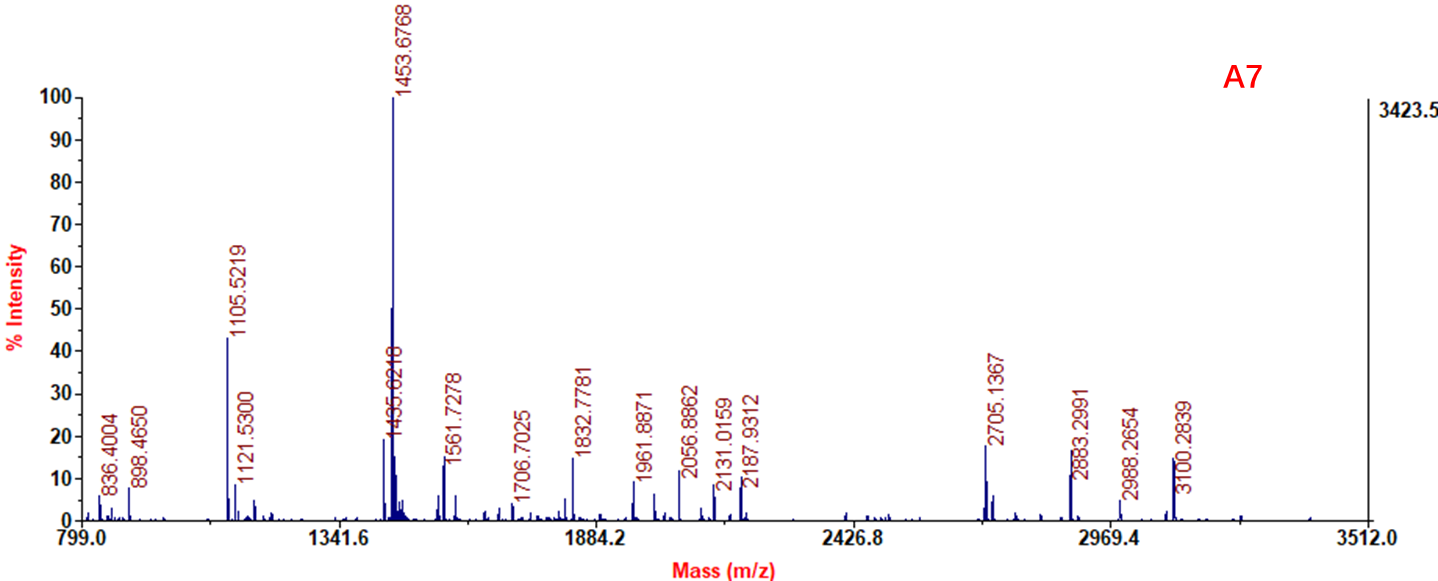

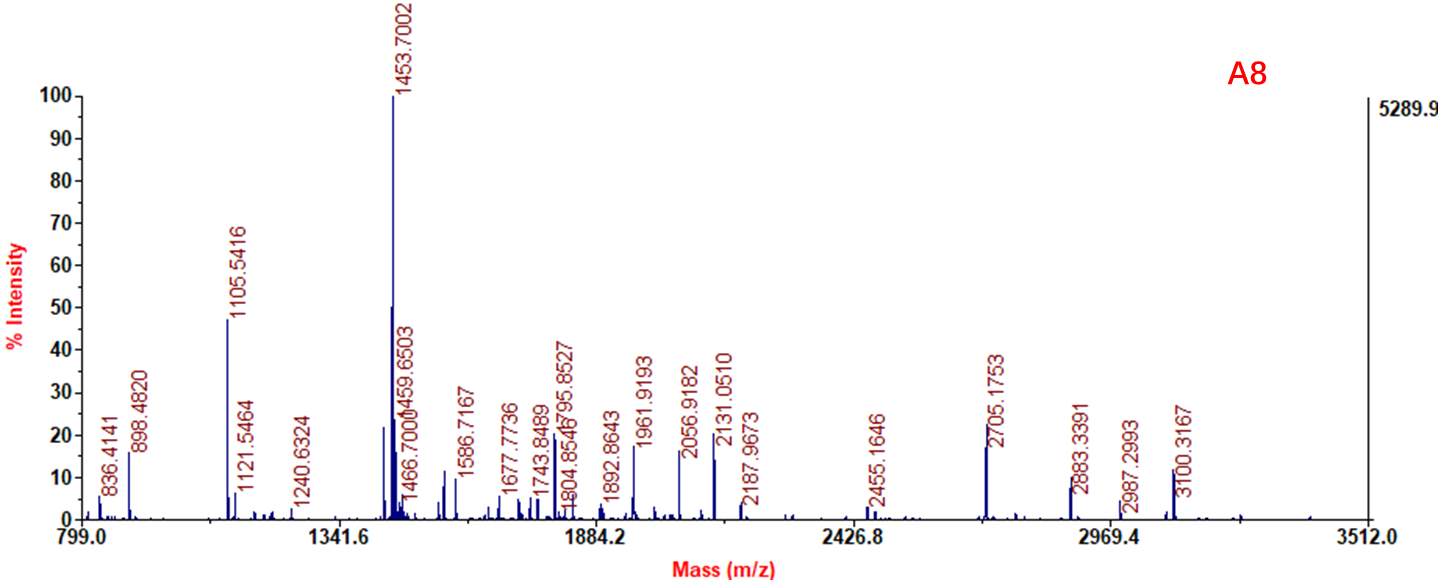

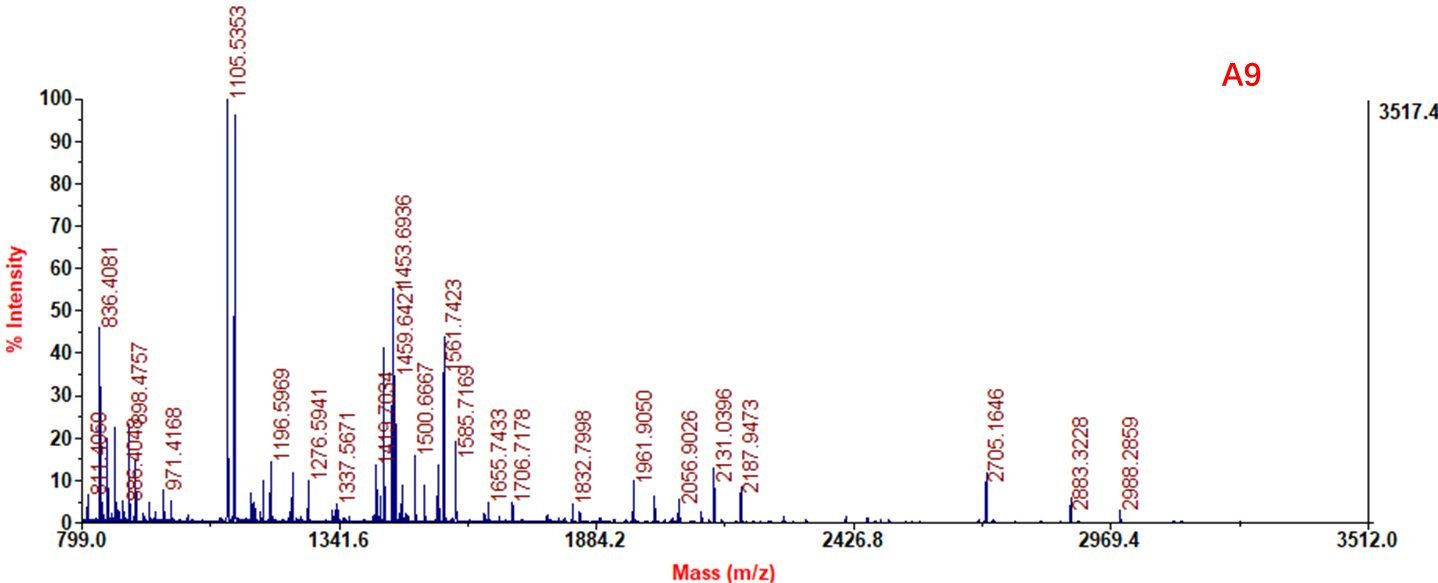

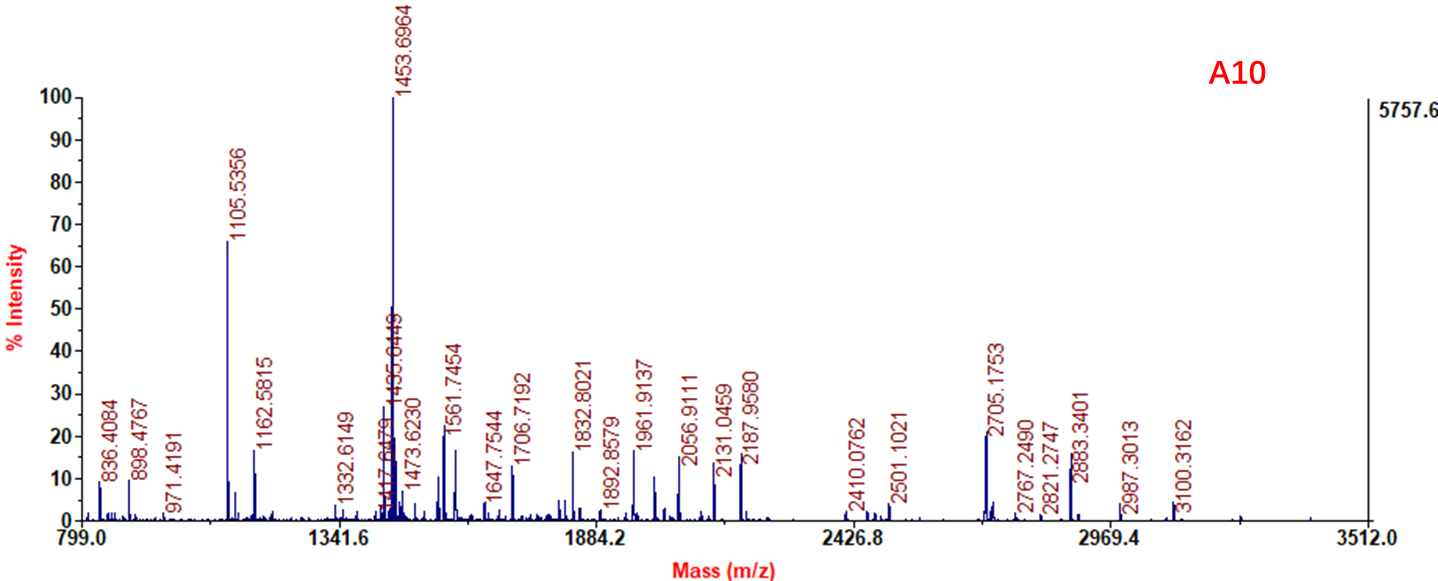

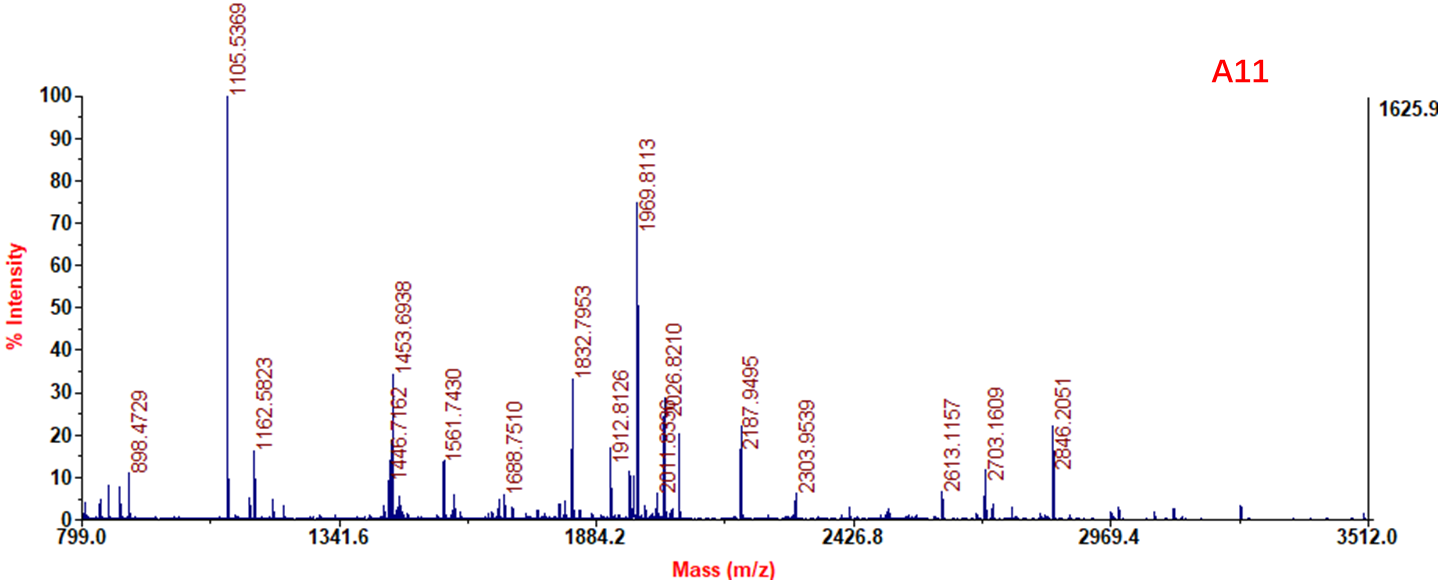

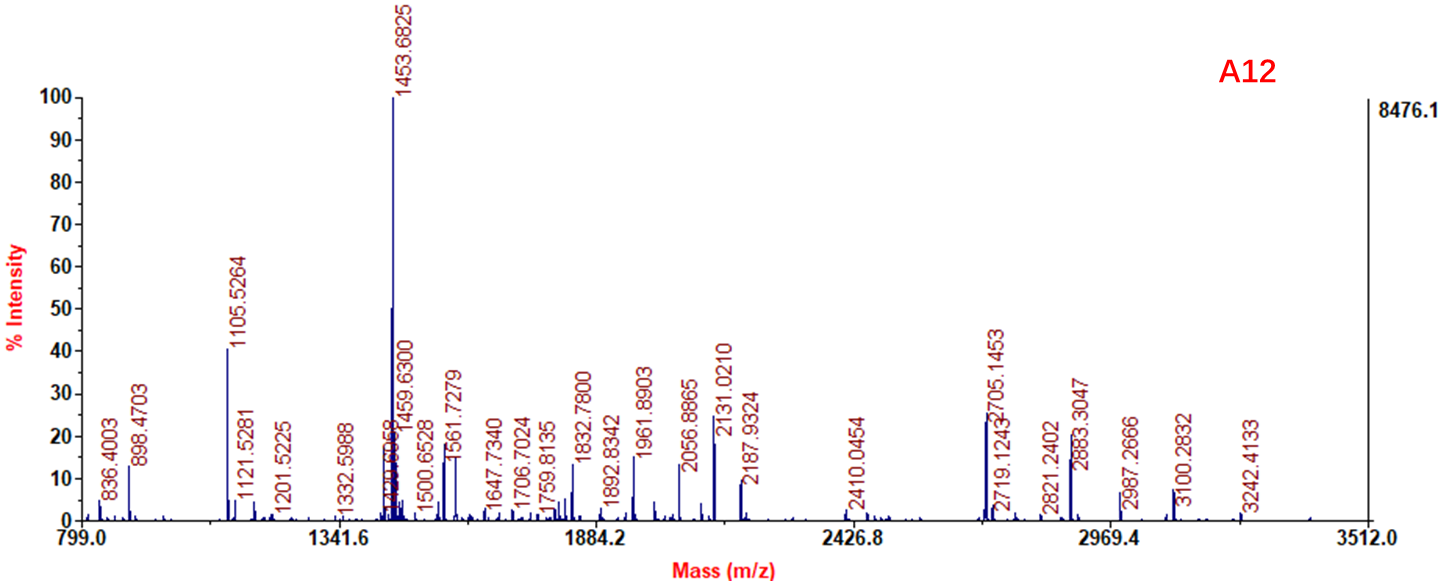

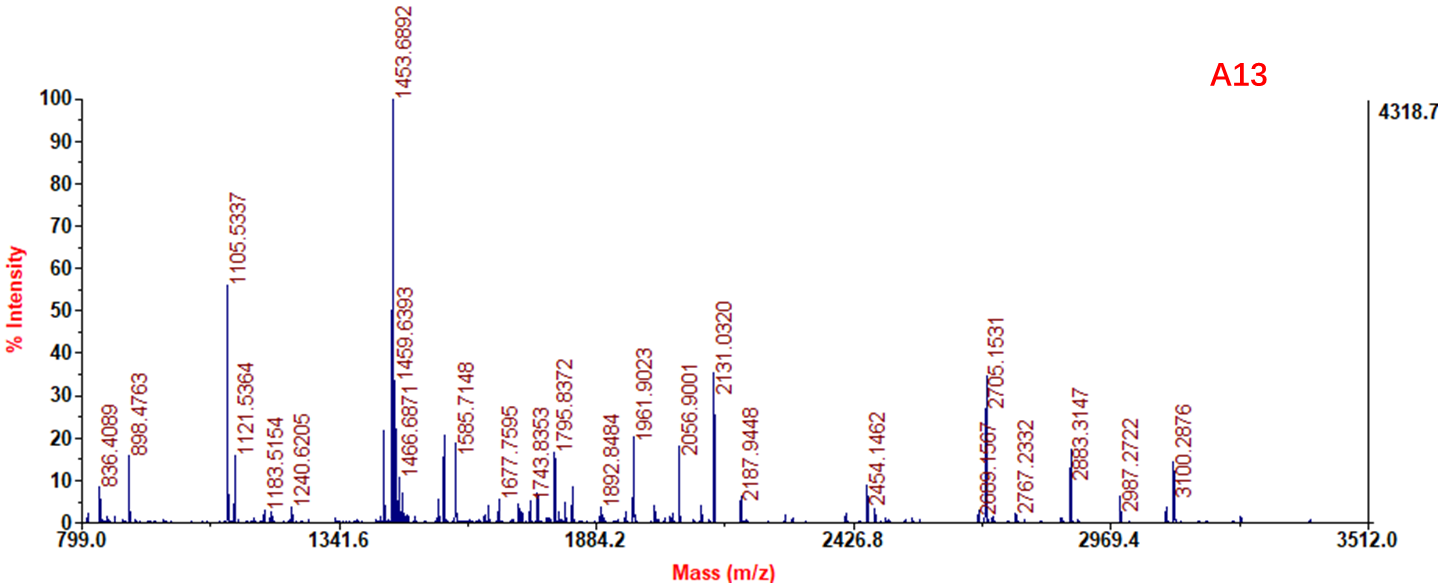

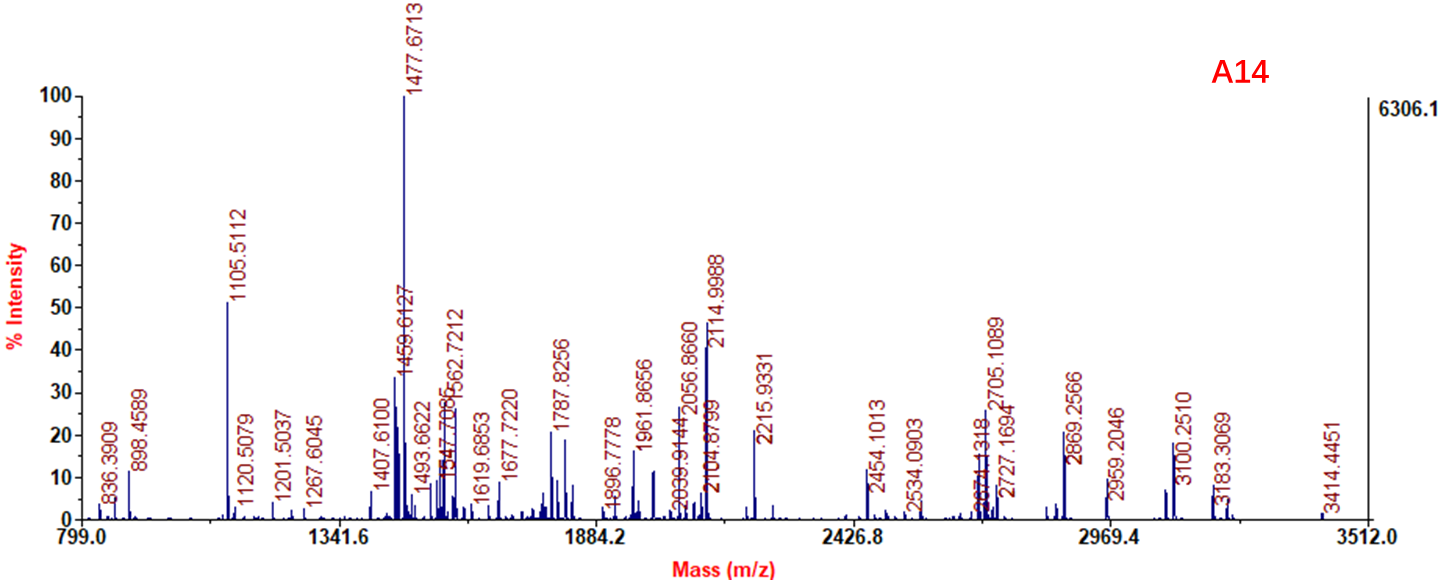

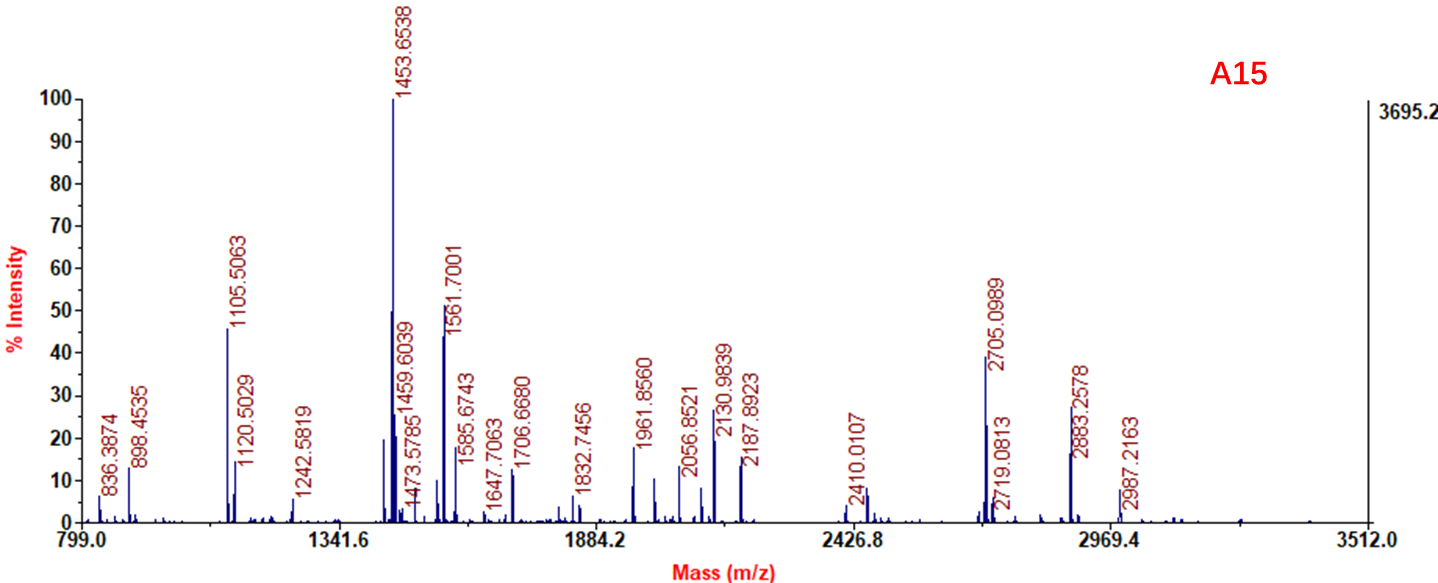

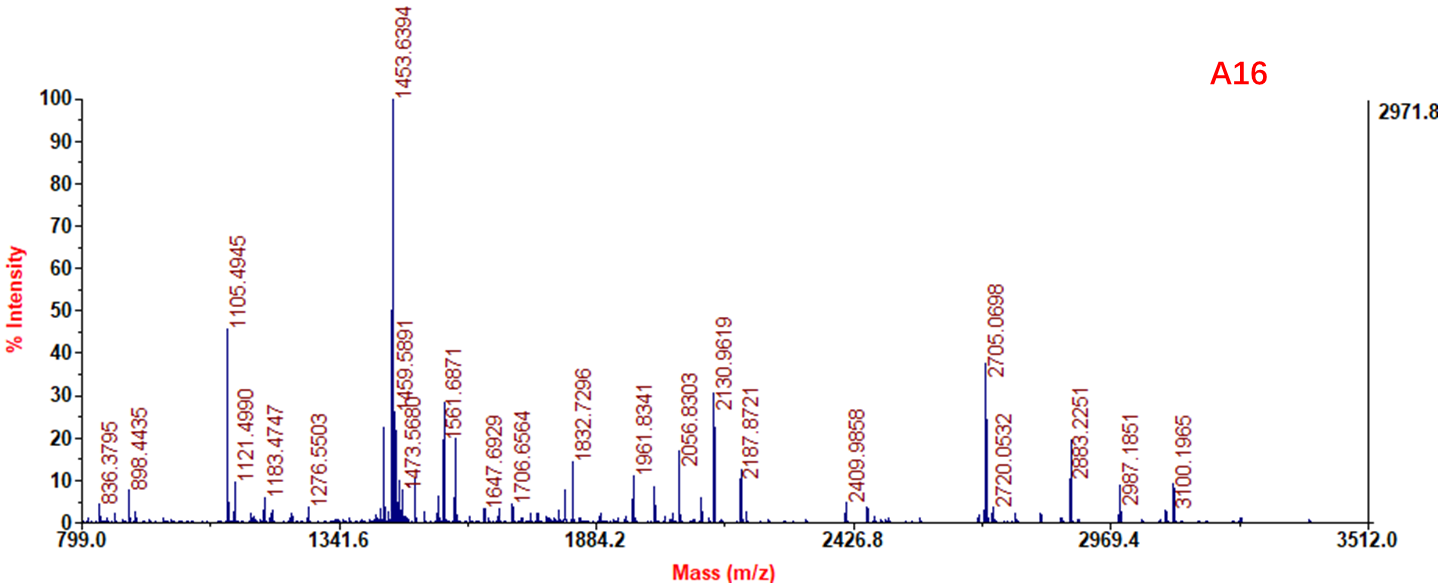

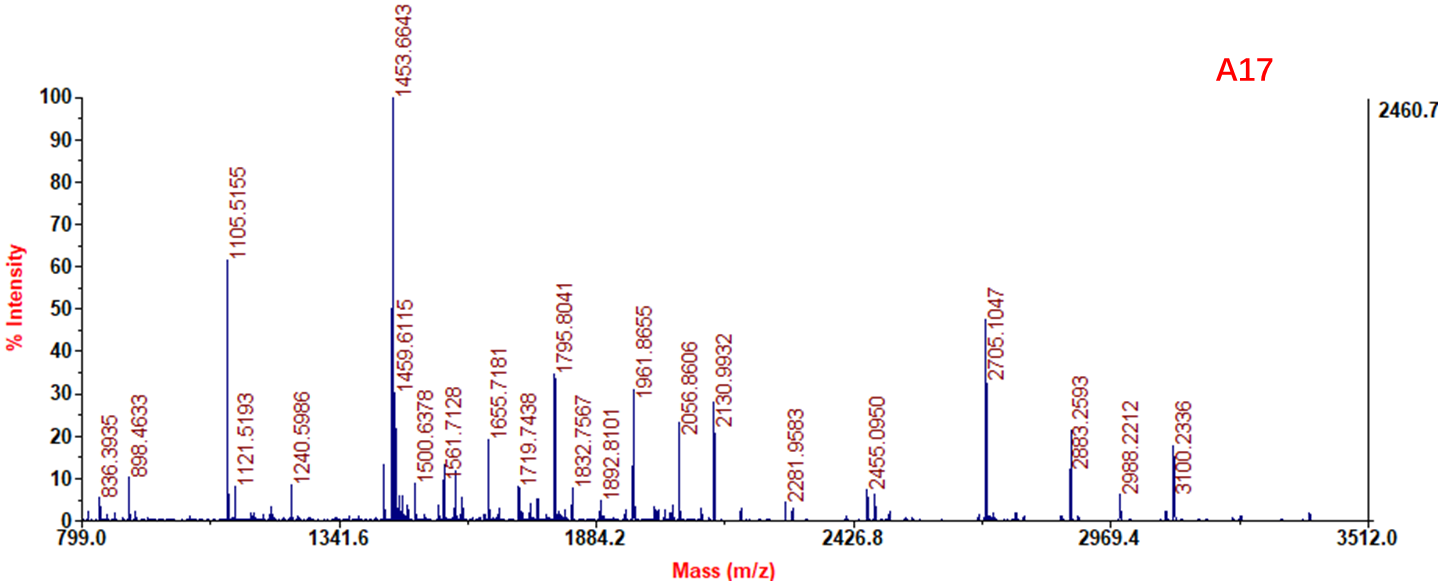

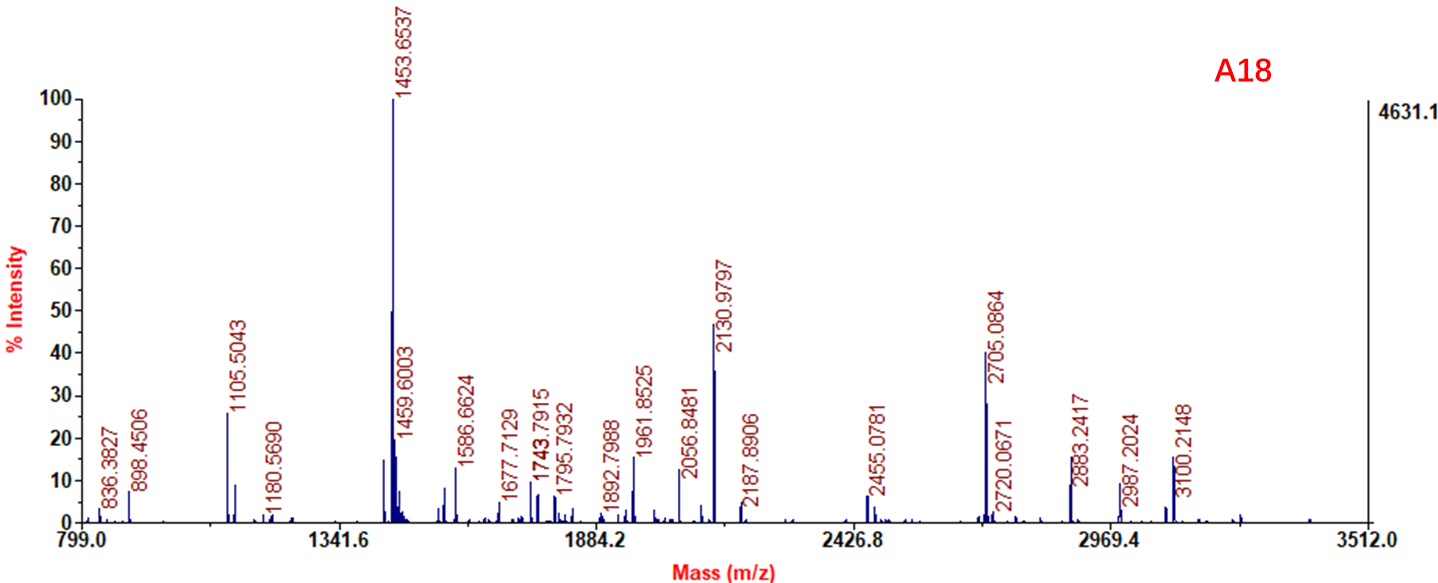

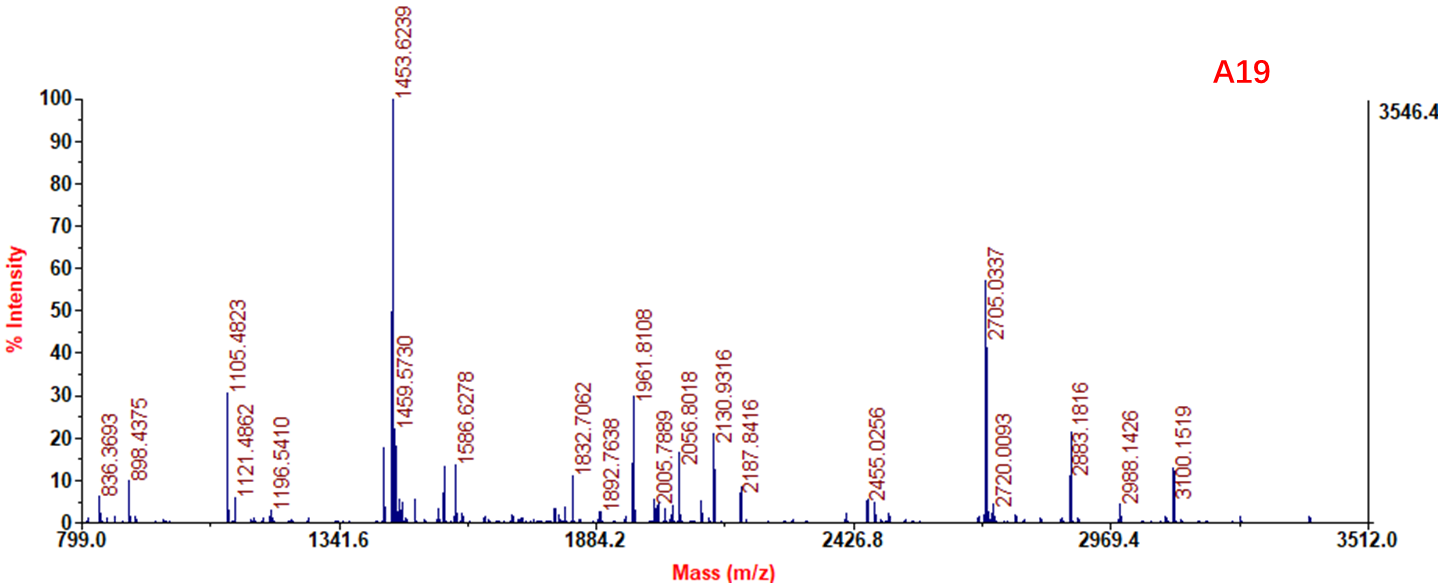


Fig. S1. MALDI-TOF spectra of the bone fragments.


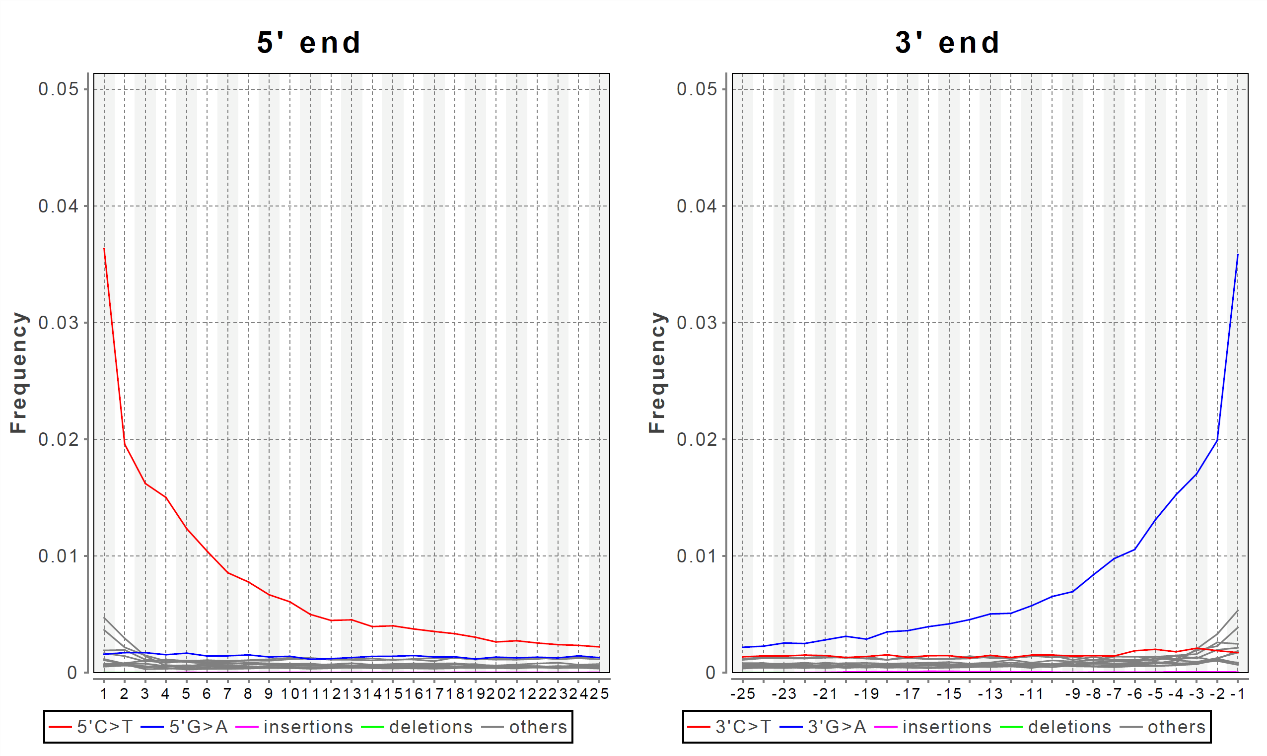


Fig. S2. DNA damage level of A14 measured by the rate of cytosine deamination-based misincorporation of bases as a function of position on reads. Red and blue lines represent 5’-end (C>T) and 3’-end (G>A) misincorporations, respectively.

Table S1. Taxonomic identification results of 19 samples by ZooMS.

| Test No. | Sample  No. | Subsample  weight (mg) | Peptide mass fingerprint data（*m/z*） | | | | | | | | | | | | ZooMS  result |
| --- | --- | --- | --- | --- | --- | --- | --- | --- | --- | --- | --- | --- | --- | --- | --- |
|  |  |  | **COL1 ɑ1**  **507-518 (P1)** | **COL1 ɑ2 978-990 (A)** | **COL1 ɑ2 978-990 (A')** | **COL1 ɑ2 484-498 (B)** | **COL1 ɑ2 502-519 (C)** | **COL1 ɑ2 292-309 (P2)** | **COL1 ɑ2 793-816（D）** | **COL1 ɑ2 454-483 (E)** | **COL1 ɑ1 585-617 (F)** | **COL1 ɑ1 585-617 (F')** | **COL1 ɑ2 757-789 (G)** | **COL1 ɑ2**  **757-789 (G')** |  |
| A1 | 2021SJ13 | 60 | 1105 |  |  | 1453 |  |  | 2131 | 2820 | 2883 |  |  |  | *Sus scrofa* |
| A2 | 2021SJ06 | 26 | 1105 |  |  | 1453 |  | 1647 | 2131 | 2820 | 2883 | 2899 |  |  | *Sus scrofa* |
| A3 | 2021SJ04 | 43 | 1105 | 1180 |  | 1453 | 1550 | 1647 | 2131 |  | 2883 | 2899 |  |  | *Sus scrofa* |
| A4 | 2021SJ01 | 47 | 1105 | 1180 |  | 1453 | 1550 |  | 2131 |  | 2883 | 2899 |  |  | *Sus scrofa* |
| A5 | 2021SJ08 | 69 | 1105 |  |  | 1453 | 1550 | 1647 | 2131 |  | 2883 | 2899 |  | 3033 | *Sus scrofa* |
| A6 | 2021SJ02 | 36 |  |  | 1319 | 1463 |  |  |  | 2212 | 2252 |  |  | 3113 | *Aves* |
| A7 | 2021SJ03 | 49 | 1105 |  |  | 1453 | 1550 | 1647 | 2131 |  | 2883 | 2899 |  |  | *Sus scrofa* |
| A8 | 2021SJ07-1 | 39 | 1105 |  |  | 1453 |  | 1647 | 2131 |  | 2883 | 2899 |  |  | *Sus scrofa* |
| A9 | 2021SJ05 | 42 | 1105 | 1180 | 1196 | 1453 | 1550 |  | 2131 |  | 2883 |  |  |  | *Sus scrofa* |
| A10 | 2021SJ09 | 42 | 1105 |  |  | 1453 | 1550 | 1647 | 2131 |  | 2883 | 2899 |  |  | *Sus scrofa* |
| A11 | 2021SJ10 | 58 | 1105 |  |  | 1453 |  |  |  |  |  |  |  |  | Fail |
| A12 | 2021SJ11 | 37 | 1105 |  |  | 1453 | 1550 | 1647 | 2131 |  | 2883 | 2899 |  | 3033 | *Sus scrofa* |
| A13 | 2021SJ12-1 | 46 | 1105 |  |  | 1453 | 1550 | 1647 | 2131 |  | 2883 | 2899 |  |  | *Sus scrofa* |
| A14 | 2021SJ12-2 | 77 | 1105 |  |  | 1477 | 1580 | 1619 | 2115 | 2832 | 2869 |  |  |  | *Homo sapiens* |
| A15 | 2021SJ12-3 | 45 | 1105 |  |  | 1453 |  | 1647 | 2131 | 2820 | 2883 | 2899 |  |  | *Sus scrofa* |
| A16 | 2021SJ12-4 | 53 | 1105 |  |  | 1453 |  | 1647 | 2131 | 2820 | 2883 | 2899 |  | 3033 | *Sus scrofa* |
| A17 | 2021SJ12-5 | 32 | 1105 | 1180 |  | 1453 | 1550 | 1647 | 2131 |  | 2883 | 2899 |  |  | *Sus scrofa* |
| A18 | 2021SJ07-2 | 40 | 1105 | 1180 |  | 1453 |  |  | 2131 | 2820 | 2883 | 2899 |  |  | *Sus scrofa* |
| A19 | 2021SJ12-6 | 43 | 1105 | 1180 | 1196 | 1453 | 1550 |  | 2131 | 2820 | 2883 | 2899 |  |  | *Sus scrofa* |

Table S2. Results of mtDNA HVR fragments extracted from bone remain of A14 and blood samples of the putative parents.

| Sample | Mutation sites in mitochondrial HVR | | | | | | | | | | |
| --- | --- | --- | --- | --- | --- | --- | --- | --- | --- | --- | --- |
|  | 16086 | 16136 | 16183 | 16184 | 16189 | 16223 | 16274 | 16309 | 16311 | 16362 |  |
| rCRS | T | T | G | C | T | C | C | A | T | T |  |
| A14 | T | T | G | T | T | T | C | A | C | C |  |
| pM | T | T | G | T | T | T | C | A | C | C |  |
| pF | C | C | C | C | C | - | T | G | T | C |  |

*Notes*：The table lists the mutated sites of mitochondrial HVR-I sequences detected in the three specimens compared with Cambridge reference sequence (rCRS)[8]

Table S3. Results of Y chromosome haplogroup analysis of A14 and pF.

| Haplogroups | Covered SNPs on the diagnostic positions of ISOGG database | |
| --- | --- | --- |
|  | A14 | pF |
| O2a2b | M1690 | F130, M1578, E303, M1585, P164, M1607, FGC16793, E488, FGC16799, CTS11109, M1729 |
| O2a2b1 | CTS11580 | M1519.1, M1546, M9120, CTS7245, M1665, CTS8881, M1691, ACT533 |
| O2a2b1a | n/a | n/a |
| O2a2b1a1 | E284 | M1516, E286, M1636, F14615, F649, F14692, CTS12991, F7810 |
| O2a2b1a1a | M1545, CTS2810, Y9142, F363 | F5, Z25787, M1521, F37, M1542, E273, M1545, F127, F139, M1567, M1591, M1597, CTS4497, M1622, CTS6104, M1647, F484, Y9139, Y9142, E530, M1704, CTS10672, CTS11637, CTS1377, F363 |
| O2a2b1a1a5 | M1532, M1726 | CTS7316 |
| O2a2b1a1a5b | n/a | F16367, A9457 |

**References:**

[1] Ning C, Wang CC, Gao S, et al. (2019) Ancient Genomes Reveal Yamnaya-Related Ancestry and a Potential Source of Indo-European Speakers in Iron Age Tianshan. Curr. Biol. 29: 2526-2532 e4 <http://doi.org/10.1016/j.cub.2019.06.044>.

[2] Peltzer A, Jager G, Herbig A, Seitz A, Kniep C, Krause J, Nieselt K (2016) EAGER: efficient ancient genome reconstruction. Genome Biol. 17: 60 <http://doi.org/10.1186/s13059-016-0918-z>.

[3] Schubert M, Lindgreen S, Orlando L (2016) AdapterRemoval v2: rapid adapter trimming, identification, and read merging. BMC Res. Notes 9: 88 <http://doi.org/10.1186/s13104-016-1900-2>.

[4] Li H, Handsaker B, Wysoker A, Fennell T, Ruan J, Homer N, Marth G, Abecasis G, Durbin R, Genome Project Data Processing S (2009) The Sequence Alignment/Map format and SAMtools. Bioinformatics 25: 2078-9 <http://doi.org/10.1093/bioinformatics/btp352>.

[5] Patterson N, Moorjani P, Luo Y, Mallick S, Rohland N, Zhan Y, Genschoreck T, Webster T, Reich D (2012) Ancient admixture in human history. Genetics 192: 1065-93 <http://doi.org/10.1534/genetics.112.145037>.

[6] Kennett DJ, Plog S, R. J. George, et al. (2017) Archaeogenomic evidence reveals prehistoric matrilineal dynasty. Nat. Commun. 8: 14115 <http://doi.org/10.1038/ncomms14115>.

[7] Jeong C, Wikin S, Amgalantugs T, et al. (2018) Bronze Age population dynamics and the rise of dairy pastoralism on the eastern Eurasian steppe. Proc. Natl. Acad. Sci. U. S. A. 115: E11248-E11255 <http://doi.org/10.1073/pnas.1813608115>.

[8] Andrews RM, Kubacka I, Chinnery PF, Lightowlers RN, Turnbull DM (1999) Reanalysis and revision of the Cambridge reference sequence for human mitochondrial DNA. Nat. Genet. 23: 147 <http://doi.org/10.1038/13779>.
